# An early complication in the donor site of the medial sural artery perforator flap: necrosis of the medial head of gastrocnemius

**DOI:** 10.1080/23320885.2019.1591279

**Published:** 2019-05-10

**Authors:** Hui-Ju Tsou, Chih-Peng Tu, Yu-Fan Chen, Wen-Teng Yao

**Affiliations:** Division of Plastic Surgery, Department of Surgery, MacKay Memorial Hospital, Taipei, Taiwan

**Keywords:** MSAP flap, medial head of gastrocnemius, early donor site complication

## Abstract

The relatively new medial sural artery perforator flap is increasingly being used for reconstruction. However, muscle necrosis of the medial head of gastrocnemius after MSAP flap harvest is a previously unnoticed early complication of the donor site. We present two cases of MSAP flap reconstruction that developed this early complication.

## Introduction

The medial sural artery perforator (MSAP) flap is an alternative flap option that is increasingly being used for reconstruction since the past decade. It was formally described in 2001 by Cavadas et al. [1]. It had been successfully used in soft tissue reconstruction of the hand [2,3] and was first used in head and neck reconstruction by Kao et al. [4]. This relatively new perforator flap has many advantages and drawbacks that have been well described [5,6]. Yet, few studies focus on the early donor site complications after MSAP flap harvest. Herein, we report two cases of reconstruction with an MSAP flap, both having previously unnoticed early complications of muscle necrosis in the donor site.

## Case reports

### Case 1

A 33-year-old healthy man presented with crushing injury to the right hand caused by a conveyor machine. A contact burn and crush injury in his right dorsal hand were noted. After escharotomy and debridement, a 13 × 7 cm^2^ skin defect in the dorsal first to third metacarpal area, intrinsic muscle loss, extensor tendon rupture, and bone exposure were noted.

We used a 15 × 10 cm^2^ free MSAP flap (number of perforators: 2, 10 cm & 11 cm to popliteal crease; length of pedicle: 11 cm) from his left leg for defect reconstruction. An end-to-end microanastomosis was successfully performed to join the radial vessels with the medial sural artery and its venae comitantes. A palmaris longus tendon from the right hand was grafted for extensor pollicis longus and third extensor digitorum communis tendon reconstruction. The donor site was covered with a split-thickness skin graft (STSG), approximately 180 cm^2^, from his ipsilateral thigh. Negative-pressure wound therapy (NPWT) was then applied to his donor site postoperatively.

**Figure 1.**
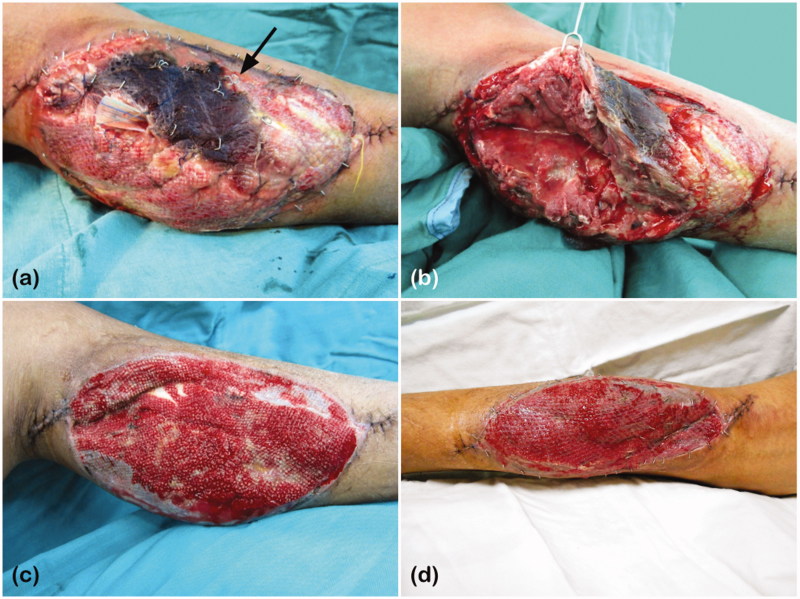
(a) Necrosis of the medial head of gastrocnemius on the left leg, indicated by the black arrow: loss of the skin graft over the area of muscle necrosis; (b) Pus formation was also noted under the necrotic muscle. (c) The wound had granulated well for STSG coverage. (d) Three days after STSG coverage and NPWT application.

However, five days after the flap reconstruction surgery, necrosis of the medial head of gastrocnemius muscle and abscess at the donor site were noted. The overlying skin graft was also lost (Figure 1a and 1b). Therefore, necrotic muscle and tissue were debrided twice, and the NPWT was re-applied. After the wound had well granulated (Figure 1c), the defect was covered with STSG, approximately 140 cm^2^ from his left thigh, for the second time. NPWT was used for 3 days postoperatively (Figure 1d).

In the outpatient follow-up, the wound healed well 5 weeks after the second STSG coverage. The patient reported no altered sensation at the donor site and had no gait problems.

### Case 2

A 45-year-old man with a medical history of coronary artery disease, hypertension, and type II diabetes mellitus was diagnosed with right buccal squamous cell carcinoma (cT3N1Mb, stage III). After wide excision, a 12 × 9 cm^2^ MSAP flap (number of perforators: 2, 8 cm & 12 cm to popliteal crease; length of pedicle: 13 cm) was harvested for reconstruction. The medial sural artery was anastomosed to the right superior thyroid artery in an end-to-end manner, and the concomitant vein was anastomosed to the right internal jugular vein by end-to-side anastomosis. The donor site was covered with STSG, approximately 150 cm^2^, from his ipsilateral thigh.

**Figure 2.**
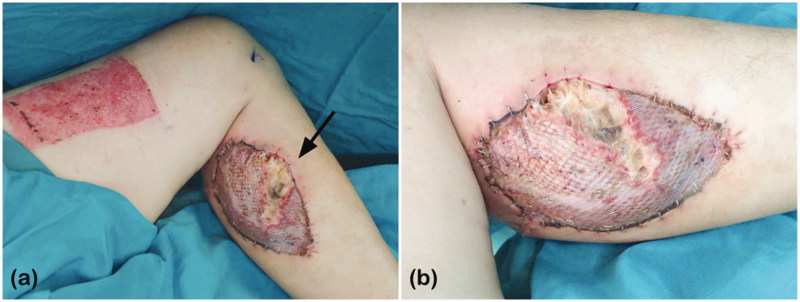
(a) Partial loss of the skin graft and (b) necrotic underlying muscle of the medial gastrocnemius at the donor site, indicated by the black arrow.

However, poor healing of the skin graft at the donor site was noted 9 days later with underlying gastrocnemius medial head muscle necrosis (Figure 2). Debridement was then performed, and the wound was allowed to heal by secondary intention owing to its small defect size. Two months after the debridement, the wound healed well. The patient had no paresthaesia at the donor site or problem walking.

## Discussion

The gastrocnemius muscle is now considered a Mathes and Nahai type II muscle [7,8]. The proximal part of the muscle is supplied by the medial and lateral sural arteries, whereas the distal part of the muscle receives branches from the posterior tibial artery and the peroneal artery. In harvesting the MSAP flap, the vascular pedicle is dissected from the medial sural artery, which originates from the popliteal vessels. Thus, even if there are small branches supplying the medial head of gastrocnemius muscle, which take off before the medial sural artery enters the muscle [7], they will be sacrificed. However, the medial gastrocnemius muscle could survive even after division of medial sural artery pedicle, owing to adjacent angiosomes. Some studies suggest that the possibility of muscle ischaemia after sacrifice of even the entire medial sural artery should thus be remote [1,2,9].

Dusseldorp et al. recently defined a new classification of the intra-muscular branching pattern of the medial sural artery [10]. There are four types: type I of a single branch, type IIA of dual branching with high take-off point above the tibial plateau, type IIB of dual branching with low take-off point below the tibial plateau, and type III of three or more branches. In type IIA, one of the dual-branch that is not dissected during flap harvest could be preserved due to the high take-off point. However, in type IIB, due to the low take-off point, the other branch that is not dissected would be sacrificed as well if the pedicle is dissected up to the origin of the medial sural artery. In the latter case, as in type I branching pattern, once the pedicle is elevated, the proximal blood supply of the medial gastrocnemius muscle is compromised. Therefore, the remaining blood supply from adjacent angiosome becomes extraordinarily important.

The existence of arterial cross-supply between the medial and lateral heads of gastrocnemius muscle has previously been demonstrated. Each head could be vascularised solely from the contralateral one [11]. However, in harvesting the MSAP flap, the vasculature from the lateral sural artery through arterial cross-supply could probably be completely transected as we dissect along the pedicle, especially when the pedicle takes a deep route. The gastrocnemius head medial to the dissected pedicle would then be isolated from the blood supply from the lateral side.

The small branches supplying the medial head of gastrocnemius muscle will also be sacrificed if the pedicle is dissected up to the origin of the medial sural artery. We suppose that in the cases of type I and type IIB, the medial head of gastrocnemius muscle, especially the proximal part, would be deprived of its blood supply after flap elevation. Thus, it would require adjacent angiosome, especially from the posterior tibial artery, for blood supply. The proximal part of the medial head, which is farthest away from the distal source of blood supply, would be prone to ischaemia and following necrosis, which may be what had happened in our cases.

However, not every case we have done using MSAP flap for reconstruction has this early complication. In the most common type [10], type IIA that has a high take-off point, we can successfully harvest the flap with the more superficial intra-muscular branch as the pedicle, and the remaining deeper branch could supply the proximal part of medial gastrocnemius muscle without being interrupted.

In our cases, pre-operative computed tomographic (CT) angiography was not routinely used to foresee the intra-muscular branching pattern of the bilateral gastrocnemius muscle and to determine the optimal leg for flap harvest [10]. Therefore, if the branching pattern happened to be either type I or type IIB, the early complication of muscle necrosis of the medial head of gastrocnemius could have happened.

In addition to determining the optimal leg for flap harvest, a pre-operative CT angiogram may also give a detailed information on the depth of the pedicle and the pedicle length that could be dissected [10]. However, the cost and safety of angiography should be taken into consideration in view of the rarity of this complication. With a handheld Doppler ultrasound and experienced intramuscular dissection technique, the MSAP flap could be well designed and harvested, minimising the risk of necrosis of the medial head of gastrocnemius muscle.

## Conclusion

The medial sural artery perforator (MSAP) flap is a relatively new flap that is increasingly being used for reconstruction. However, muscle necrosis of the medial head of gastrocnemius after MSAP flap harvest is a previously unnoticed early complication of the donor site.

We suppose that muscle necrosis is less likely to occur in type IIA intramuscular branching pattern owing to its remaining blood supply. However, the early complication could occur after MSAP flap harvest with either type I or type IIB branching pattern.

If possible, pre-operative CT angiography could help determine the optimal leg for flap harvest, but its cost and safety should be well considered in view of the rarity of this complication. When combining experienced intramuscular dissection technique with handheld Doppler ultrasound, the MSAP flap could be well designed and harvested, minimising the risk of such early complication of muscle necrosis.
